# Involvement of NADH Oxidase in Competition and Endocarditis Virulence in Streptococcus sanguinis

**DOI:** 10.1128/IAI.01203-15

**Published:** 2016-04-22

**Authors:** Xiuchun Ge, Yang Yu, Min Zhang, Lei Chen, Weihua Chen, Fadi Elrami, Fanxiang Kong, Todd Kitten, Ping Xu

**Affiliations:** a Philips Institute for Oral Health Research, Virginia Commonwealth University, Richmond, Virginia, USA; b State Key Laboratory of Lake Science and Environment, Nanjing Institute of Geography and Limnology, Chinese Academy of Science, Nanjing, China; c Department of Microbiology and Immunology, Virginia Commonwealth University, Richmond, Virginia, USA; Tufts University School of Medicine

## Abstract

Here, we report for the first time that the Streptococcus sanguinis *nox* gene encoding NADH oxidase is involved in both competition with Streptococcus mutans and virulence for infective endocarditis. An S. sanguinis *nox* mutant was found to fail to inhibit the growth of Streptococcus mutans under microaerobic conditions. In the presence of oxygen, the recombinant Nox protein of S. sanguinis could reduce oxygen to water and oxidize NADH to NAD^+^. The oxidation of NADH to NAD^+^ was diminished in the *nox* mutant. The *nox* mutant exhibited decreased levels of extracellular H_2_O_2_; however, the intracellular level of H_2_O_2_ in the mutant was increased. Furthermore, the virulence of the *nox* mutant was attenuated in a rabbit endocarditis model. The *nox* mutant also was shown to be more sensitive to blood killing, oxidative and acid stresses, and reduced growth in serum. Thus, NADH oxidase contributes to multiple phenotypes related to competitiveness in the oral cavity and systemic virulence.

## INTRODUCTION

NADH oxidase can catalyze the reduction of oxygen to H_2_O_2_ or H_2_O with concomitant oxidation of NADH to NAD^+^ in bacteria. During glycolysis, bacterial cells produce NADH from NAD^+^. To balance the NAD^+^/NADH ratio for maintaining glycolysis, NADH oxidase has been recognized as playing an important role in producing NAD^+^ from NADH. Yamamoto et al. (1) have proposed that NADH oxidase is involved in converting pyruvate to acetyl-coenzyme A (CoA) under aerobic conditions in Streptococcus agalactiae. There are two genes, *nox-1* and *nox-2*, encoding NADH oxidases in Streptococcus mutans (2, 3). The *nox-1* gene encodes an H_2_O_2_-forming NADH oxidase (3), whereas *nox-2* has been proposed to encode an H_2_O-forming NADH oxidase (2). However, most streptococci, including Streptococcus pneumoniae and S. agalactiae, possess orthologs of only *nox-2*. In S. agalactiae, the inactivation of *nox* was shown to reduce or eliminate growth under aerobic conditions (1), while growth was not affected by *nox* inactivation in S. pneumoniae under aerobic or anaerobic conditions (4). In S. pneumoniae and S. agalactiae, *nox* inactivation attenuates virulence in animal models (1, 4). In addition, the efficiency of competence for genetic transformation was significantly altered in a S. pneumoniae *nox* mutant (4). These data imply NADH oxidase is important for multiple biological functions in streptococci.

Infective endocarditis (IE) is a dangerous disease with a mortality rate of approximately 30% at 1 year (5). Between 2000 and 2011, the incidence of IE in the United States increased from 11 to 15 cases per 100,000 persons (6). Treatment of endocarditis is complicated. Medical treatment can involve prolonged hospitalization and often fails, necessitating the surgical replacement of infected heart valves (7–9). Antibiotic prophylaxis generally has not been recommended for invasive dental procedures for many years, and IE prophylaxis for dental procedures has been restricted to a smaller number of cardiac conditions with very high risk for adverse outcomes from IE (10). IE may be complicated by an increasing frequency of antibiotic resistance (11). The oral streptococci are common causes of IE (6, 12). The incidence of streptococcal IE in the United States rose significantly, from 26 to 42 cases per million persons, between 2000 and 2011 (6).

Streptococcus sanguinis is a normal inhabitant in the oral cavity but one of the most common pathogens of IE (13–15). It can inhibit the growth of S. mutans and is regarded as an antagonistic bacterium against S. mutans in the oral cavity (16). The production of H_2_O_2_ has been demonstrated to be responsible for the inhibition by S. sanguinis of the growth of S. mutans (16). In our previous studies, we identified several genes that are related to both competition and H_2_O_2_ production, including *spxB*, *ackA*, *spxR*, *spxA1*, and *tpk* (17, 18).

In S. sanguinis, an ortholog of *nox-2* (named *nox* in this study; SSA_1127) is present, but there is no *nox-1* ortholog. In this study, we found the *nox* gene was involved in competition with S. mutans as well as virulence for IE and examined the possible mechanisms by which the *nox* gene could affect the competition and IE.

## MATERIALS AND METHODS

### Ethics statement.

All animal experiments were handled in compliance with the U.S. Office of Laboratory Animal Welfare and U.S. Department of Agriculture guidelines, as well as institutional policies. All procedures were approved by the Virginia Commonwealth University Institutional Animal Care and Use Committee (protocol AM10030).

### Bacterial strains, growth, and antibiotics.

S. sanguinis strain SK36 and its mutants and S. mutans UA159 (Table 1) were grown in brain heart infusion (BHI) broth or agar (BD, San Jose, CA) at 37°C under microaerobic conditions (6% O_2_, 7.2% CO_2_, 7.2% H_2_, and 79.6% N_2_) as described previously (19). Antibiotics, including 500 μg/ml kanamycin and 10 μg/ml erythromycin (Fisher scientific, Pittsburgh, PA), were used for mutant construction and culture.

**TABLE 1:** Strains and primers in this study

Strain or primer	Description or sequence^*a*^	Source
S. sanguinis strains		
SK36	Human plaque isolate	Kilian et al. (37)
Δ*nox*	Kan^r^; ΔSSA_1127::*aphA-3*	This study
Δ*nox_compl*	Erm^r^; ΔSSA_1127::*erm*	This study
Δ*spxB*	Kan^r^; ΔSSA_0391::*aphA-3*	Chen et al. (17)
JFP36	Erm^r^; ΔSSA_0169::*pSerm*	Turner et al. (25)
Primers		
nox_F1	CCATCTACCGACTTGTCTGAAAC	
nox_R1	GCCATTTATTCCTCCTAGTTAGTCAACTCATAAGAATAGTCCTACCTTA	
Kan_F2	TGACTAACTAGGAGGAATAAATGGCTAAAATGAGAATAT	
Kan_R2	CATTATTCCCTCCAGGTACTAAAACAATTCATCCAGT	
nox_F3	GTTTTAGTACCTGGAGGGAATAATGATTACTCAAGCAGCTTTGAAAGC	
nox_R3	GTAGGAAATAACCAATCGGAAGAAT	
nox_compl_F1	nox_F1	
nox_compl_R1	TGTAATCACTCCTTCTCACTATTTATTTTGCTTTCAAAGCTGCTTGA	
Erm_F2	TAAATAGTGAGAAGGAGTGATTACATGAACAA	
Erm_R2	TTATTTCCTCCCGTTAAATAATAG	
nox_compl_F3	CTATTATTTAACGGGAGGAAATAAGAAAATGAGTCTGGGATAAATTTCCA	
nox_compl_R3	nox_R3	
nox_rp_F	GACGACGACAAGATCAGTAAAATCGTTGTAGTTGGTGCAA	
nox_rp_R	GAGGAGAAGCCCGGTTATTTTGCTTTCAAAGCTGCTTGA	
spxB_L	ATCACTCAACACCGTCCACTTCCA	
spxB_R	TCTTCCAAGAAGAGGCGGAATGGT	
gyrA_L	AGCTGATTGCCTTGATTGCAGAC	
gyrA_R	ATCCGCAAATTTACGCTTGACCT	

a Kan, kanamycin; Erm, erythromycin.

### Deletion and complementation of the *nox* gene.

The open reading frame (ORF) of the *nox* gene in S. sanguinis SK36 was replaced by a promoterless kanamycin cassette (*aphA-3*) as described previously (19). Briefly, three pairs of primers, nox_F1 and nox_R1, nox_F3 and nox_R3, and kan_F2 and Kan_R2 (Table 1), were used for PCR amplification of 1-kb upstream and downstream flanking regions of the *nox* gene and for the promoterless *aphA-3*, respectively. The three PCR-amplified fragments were combined by second-round PCR amplification using primers nox_F1 and nox_R3. The final linear recombinant PCR amplicon was transformed into S. sanguinis SK36 to obtain the *nox*-deleted mutant using kanamycin for selection.

The *nox* mutant was complemented by a similar strategy. Upstream sequence (1 kb) plus the ORF of the *nox* gene, the promoterless erythromycin cassette (*erm*), and 1 kb of sequence downstream of the *nox* gene were PCR amplified and then combined to obtain the recombinant PCR amplicon in which the *nox* ORF was followed by the *erm* cassette. The recombined amplicon was transformed into the *nox* mutant to obtain a complemented strain of the *nox* mutant using erythromycin for selection. The primers used are listed in Table 1.

### Determination of S. mutans inhibition by S. sanguinis.

The inhibition of S. mutans by S. sanguinis was determined as described previously (17). Briefly, cultures of S. sanguinis strains were dropped onto BHI agar plates to form spots and incubated microaerobically at 37°C. After overnight growth, S. mutans UA159 cultures were dropped near S. sanguinis spots and incubated microaerobically at 37°C for 1 day. No growth of S. mutans in the contact zone on an agar plate was viewed as the inhibition of S. mutans by S. sanguinis; otherwise, S. sanguinis was judged to have failed to inhibit S. mutans.

### Expression and purification of rNox protein.

The cloning, expression, and purification of recombinant Nox (rNox) protein of S. sanguinis in Escherichia coli was performed as described previously (20). Briefly, the S. sanguinis nox gene was PCR amplified using primers nox_rp_F and nox_rp_R (Table 1) and cloned into pET-46 Ek/LIC vector (Novagen, Madison, WI) according to the manufacturer's protocol. After being confirmed by sequencing, the plasmid was transformed and expressed in E. coli BL21(DE3)pLysS (Novagen, Madison, WI). The expressed rNox protein with N-terminal His tag was isolated using BugBuster buffer (Novagen, Madison, WI) and purified using a His · Bind column chromatography kit (Novagen, Madison, WI) as described in the manufacturer's protocols. SDS-PAGE then was performed to confirm the purified rNox protein and to estimate its purity (20). The protein was aliquoted and stored at −20°C until use.

### Enzyme assays.

To measure the oxidation of NADH to NAD^+^ by rNox, the reaction mixture containing 50 mM potassium phosphate buffer (pH 7.0), 0.1 mM β-NADH, and purified rNox was monitored at 340 nm for a decrease in NADH absorbance at room temperature. Prior to the addition of rNox, the mixture was saturated with oxygen by bubbling compressed air through the reaction mixture. One unit of enzyme activity was defined as the amount that catalyzed the oxidation of 1 μmol of NADH per min at room temperature.

To determine whether H_2_O_2_ was generated in the NADH oxidation reaction by rNox, the reaction mixture was compared to an equivalent reaction mixture containing the Bacillus licheniformis NADH oxidase protein (EMD Millipore, Billerica, MA). β-NADH (0.1 mM) was oxidized by rNox or B. licheniformis NADH oxidase protein in oxygen-saturated potassium phosphate buffer (50 mM, pH 7.0) and 0.1 mM flavin adenine dinucleotide (FAD) in a total volume of 800 μl. Following the completion of NADH oxidation, 200 μl of a solution containing 22 mg/ml 2,2-azino-bis(3-ethylbenzothiazoline-6-sulfonic acid) (ABTS) and 100 U/ml horseradish peroxidase was added (21). The mixtures then were incubated for 30 min and measured for absorbance at 725 nm. A standard curve was made using exogenous H_2_O_2_.

The NADH oxidase activity of S. sanguinis cell lysates was measured spectrophotometrically according to Yamamoto et al. (1). Briefly, S. sanguinis wild-type, mutant, and complemented cells, cultured in BHI broth up to exponential growth phase (optical density at 450 nm [OD_450_] of ∼0.8) under microaerobic conditions, were harvested and washed by centrifugation and mechanically disrupted in cold 50 mM potassium phosphate buffer (pH 7.0) with 1 mM phenylmethylsulfonyl fluoride (PMSF) using FastPrep lysing matrix B (MP Biomedicals, Solon, OH). The disrupted cell suspensions were centrifuged at 16,000 × *g* for 15 min at 4°C, and the supernatant was harvested to use for NADH oxidase activity assays. The activity was measured in the reaction mixture composed of oxygen-saturated potassium phosphate buffer, 0.1 mM β-NADH, and cell extract at room temperature by monitoring the OD_340_.

In all assays, the recombinant SSA_0375 protein, annotated as a lipoprotein transporter and prepared the same way as rNox, was used as a negative control, and protein concentrations were determined by the Bradford method (22) using bovine serum albumin as a standard. Unless otherwise stated, all chemicals were purchased from Sigma-Aldrich (St. Louis, MO).

### Determination of extracellular and intracellular H_2_O_2_ production.

To determine whether extracellular H_2_O_2_ was changed in the S. sanguinis *nox* mutant, the Amplex red assay was performed as described previously (17). Exponential cultures of S. sanguinis SK36, the *nox* mutant, and its complemented strain under microaerobic conditions were diluted 100-fold in prewarmed BHI with Amplex red (50 μM) and 0.1 U/ml horseradish peroxidase (Life Technologies, Grand Island, NY). The diluted cultures were incubated at 37°C in a FLUOstar microplate reader (BMG Technologies), and OD_560_ values were measured at 10-min intervals for 4 h.

To examine whether intracellular H_2_O_2_ was changed in the S. sanguinis *nox* mutant, the cells were isolated and disrupted for H_2_O_2_ determination. Exponential cultures of SK36, the *nox* mutant, and its complemented strain grown under microaerobic conditions were washed with ice-cold potassium phosphate buffer (50 mM, pH 7.0) with 1 mM PMSF by 4,000-rpm centrifugation at 4°C and resuspended in potassium phosphate buffer on ice. The suspended cells were disrupted using FastPrep lysing matrix B and then centrifuged at 16,000 × *g* at 4°C for 15 min. The supernatants were harvested and used for H_2_O_2_ determination. Amplex red (50 μM) and 0.1 U/ml horseradish peroxidase were added to the supernatants. The same supernatants also were assayed for pyruvate oxidase (SpxB) activity. The cell lysates were added to the reaction mixture containing oxygen-saturated potassium phosphate buffer (50 mM, pH 7.0), 0.05 mM thiamine pyrophosphate, 0.01 mM FAD, 0.97 mM MgSO_4_, 1.5 mM sodium pyruvate, Amplex red (50 μM), and 0.1 U/ml horseradish peroxidase. The reaction mixture without sodium pyruvate was set as the baseline. All reactions in the same plate were monitored at a wavelength of 560 nm at 37°C in a FLUOstar microplate plate reader.

### qRT-PCR.

To analyze the expression of *spxB* by quantitative RT-PCR (qRT-PCR), S. sanguinis SK36, the *nox* mutant, and the complemented strain cells were cultured microaerobically at 37°C in BHI broth. At the exponential growth phase (OD_450_ of ∼0.8), RNAprotect bacterial reagent (Qiagen, Valencia, CA) was added to the cultures (2:1) to stabilize the RNA, and then cells were harvested by centrifugation at 4,000 × *g* for 15 min. RNA from the sample cells was isolated through lysozyme lysis, mechanical disruption with FastPrep lysing matrix B, and purification with an RNeasy minikit (Qiagen, Valencia, CA) as described in the manufacturer's protocol. DNA was removed by treatment in columns with DNase I during purification. In the reverse transcription reaction, first-strand cDNA was synthesized in a 20 μl-reaction mixture containing 4 μl of 5× first-strand buffer, 100 ng RNA, 1.5 μg random primers, 1 μl of 10 mM deoxynucleoside triphosphate (dNTP) mix, 1 μl of 0.1 M dithiothreitol (DTT), 1 μl RNaseOUT recombinant RNase inhibitor (40 U/μl), and 1 μl of SuperScript III reverse transcriptase (200 U/μl) by following the manufacturer's protocol (Life Technologies, Grand Island, NY). The reaction without reverse transcriptase was conducted in parallel as a control for possible DNA contamination. The qRT-PCR was composed of 5 μl SYBR green PCR master mix (Life Technologies, Grand Island, NY), 10 pmol each of paired primers spxB_L and spxB_R (Table 1), and 1 μl of 50-fold-diluted cDNA template. The reaction was performed at 50°C for 2 min, 95°C for 10 min, and 40 cycles of 95°C for 15 s and 60°C for 1 min using an Applied Biosystems 7500 Fast real-time PCR system (Life Technologies, Grand Island, NY), followed by dissociation curve analysis. The housekeeping gene *gyrA*, with primers gyrA_L and gyrA_R (Table 1), was used as a normalization control. The specificity of the primers for the genes was determined by melting profiles from the dissociation curve analyses and agarose gel electrophoreses of the qRT-PCR products (23). The standard curves were prepared using serial dilutions of SK36 chromosomal DNA as templates and used for qRT-PCR efficiency analyses and quantification.

### CI in a rabbit endocarditis model and *in vitro*.

A competitive index (CI) of the *nox* mutant to the wild type was used to evaluate the virulence of the *nox* mutant in a rabbit endocarditis model, which was described previously (24). Briefly, equal amounts of overnight-cultured *nox* mutant and JFP36, a derivative of the wild-type strain SK36 with an erythromycin-resistant cassette inserted in the SSA_0619 locus and demonstrating the same virulence as the wild type with no polar effects (25), was diluted 10-fold in BHI and incubated at 37°C for 3 h. After washing with sterile phosphate-buffered saline (PBS), the bacterial cells were adjusted to ∼2 × 10^8^ CFU/ml, and 0.5 ml cells was inoculated into catheterized New Zealand White rabbits in triplicate. The vegetations on rabbit heart valves were collected the next day. The vegetations were homogenized, serially diluted in PBS, and spread on BHI plates supplemented with erythromycin or kanamycin for bacterial enumeration. The CI was determined as the Δ*nox*/JFP36 ratio of the output CFU divided by the Δ*nox*/JFP36 ratio of the inoculum. For *in vitro* CI assay for the examination of the competitive growth of the *nox* mutant and JFP36, the inoculum described above was diluted 1,000-fold in BHI and incubated microaerobically overnight at 37°C. Cells were serially diluted and enumerated as described above. The *in vitro* CI was determined as the mutant/wild-type ratio of the overnight culture divided by the mutant/JFP36 ratio of the inoculum.

### Blood killing.

Overnight microaerobically cultured *nox* mutant and JFP36 cells were diluted 10-fold in BHI and incubated at 37°C for 3 h. Equal volumes of *nox* mutant and JFP36 were mixed together, washed with Hanks' balanced salt solution (HBSS) buffer, and resuspended in HBSS buffer to approximately 2 × 10^8^ CFU/ml. The suspension was mixed 1:9 with human fresh blood (Virginia Blood Service) and incubated at 37°C with rotary shaking at 250 rpm. After 0, 45, and 90 min of incubation, the mixture was serially diluted in sterile distilled H_2_O and spread on erythromycin- or kanamycin-containing BHI agar plates for CFU counting. The bacterial survival was expressed as the Δ*nox*/JFP36 ratio of CFU at treatment time divided by the Δ*nox*/JFP ratio of CFU at time zero.

### Serum growth assay.

Overnight cultures of S. sanguinis SK36, the *nox* mutant, and its complemented strain were diluted 100-fold in prewarmed BHI broth and incubated at 37°C under microaerobic conditions. At exponential growth phase (OD_450_ of ∼0.8), cells were harvested and washed with sterile PBS by centrifugation. The resuspended cells then were diluted 1:10,000 in human serum (Fisher Scientific, Pittsburgh, PA) and cultured microaerobically at 37°C overnight. The cells from overnight culture were serially diluted in PBS, spread on BHI agar plates, and enumerated after a 2-day incubation.

### H_2_O_2_ and acid sensitivity assay.

As described above, exponential-growth-phase cells of S. sanguinis SK36, the *nox* mutant, and its complemented strain cultured microaerobically in BHI broth were collected and washed with sterile PBS for stress treatment. The cell suspension was treated with 20 mM H_2_O_2_ (Fisher Scientific, Pittsburgh, PA) at 37°C for 30 and 60 min or with sterile 50 mM glycine, pH 4.0, for 60 min and then serially diluted in PBS. The serial dilutions were plated on BHI agar, and the colonies were counted after a 2-day incubation. The bacterial survival was expressed by the percentage of CFU in the treated versus untreated cells.

### Statistical analysis.

All data were obtained in at least biological triplicate. Student's *t* test was used for NADH oxidase activity analysis of rNox. For data on qRT-PCR and CI, one-sample *t* test was applied to analyze the values of mutant or complemented mutant compared to a value of 1. Other data were statistically analyzed by analysis of variance (ANOVA) with *post hoc* Tukey's honestly significant different (HSD) test. The significance was set as a *P* value of <0.05.

## RESULTS

### Diminishment of competition in the S. sanguinis *nox* mutant.

**FIG 1:**
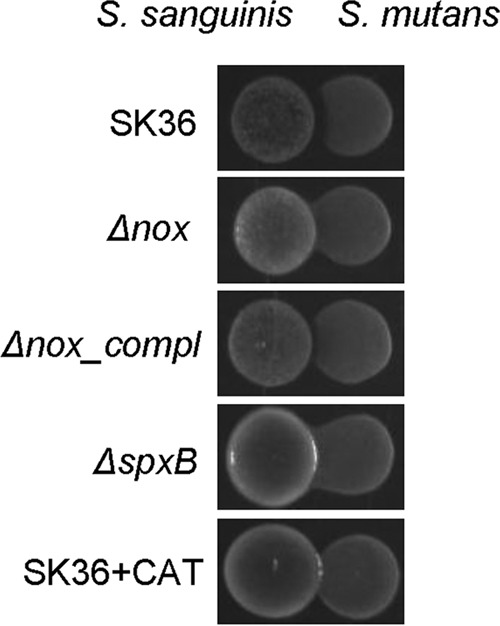
Diminished inhibition of S. mutans in the *nox* mutant. Δ*nox*, the *nox* mutant; Δ*nox_compl*, the complemented strain of the *nox* mutant; Δ*spxB*, the *spxB* mutant; CAT, catalase spread on plate prior to inoculation at ∼880 U cm^−2^.

To examine whether the *nox* gene is involved in competition, the effect of the *nox* deletion on the inhibition of S. mutans was assessed on BHI agar plates under microaerobic conditions. The result showed the wild-type and *nox*-complemented strains inhibited S. mutans at the contact zone on agar plates, but the *nox* mutant failed to inhibit S. mutans (Fig. 1). The mutation of the *nox* gene in SK36 produced an effect that was similar to that of catalase addition (Fig. 1). These data suggest that the *nox* gene is involved in the competition of S. sanguinis with S. mutans, and that the decreased H_2_O_2_ secretion is responsible for the diminished inhibition of S. mutans. To confirm the complementary result, we also examined the competition of *nox* gene neighboring mutants with S. mutans by comparing the upstream gene mutants from SSA_1114 to SSA_1126 and downstream gene mutants from SSA_1128 to SSA_1130. We did not find the loss of competition in any of these mutants, suggesting a lack of polar effect (data not shown).

### H_2_O-forming NADH oxidase activity of the rNox protein.

**FIG 2:**
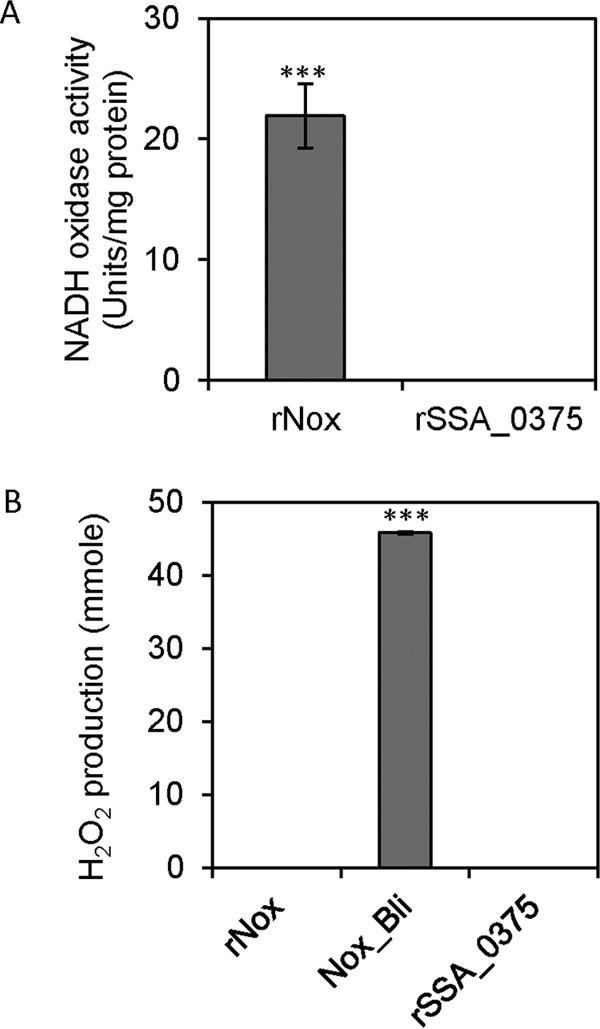
NADH oxidase activity and assessment of H_2_O_2_ production by the Nox protein. (A) NADH oxidase activities of rNox and recombinant SSA_0375 (rSSA_0375) protein (a negative control). (B) Hydrogen peroxide production of rNox, Bacillus licheniformis NADH oxidase protein (Nox_Bli), and rSSA_0375 protein. ***, *P* < 0.001. Data were obtained at least in biological triplicate.

To determine whether Nox directly generates H_2_O_2_, we prepared rNox protein in E. coli by cloning the S. sanguinis *nox* gene into vector pET-46 EK/LIC and introducing the subsequent plasmid into E. coli BL21(DE3)pLysS for expression. The NADH oxidase activity of the purified rNox protein was assayed under aerobic conditions. The result showed that the rNox protein oxidized NADH to NAD^+^, unlike the negative-control recombinant SSA_0375 protein (Fig. 2A). However, this protein did not generate H_2_O_2_, unlike the positive-control B. licheniformis NADH oxidase protein, which produced abundant H_2_O_2_ from oxygen (Fig. 2B). These data indicate that the rNox protein has the activity of an H_2_O-forming NADH oxidase under aerobic conditions, which is consistent with the annotation of the S. sanguinis *nox* gene as encoding an H_2_O-forming NADH dehydrogenase.

### Diminishment of NADH oxidase activity in the *nox* mutant.

**FIG 3:**
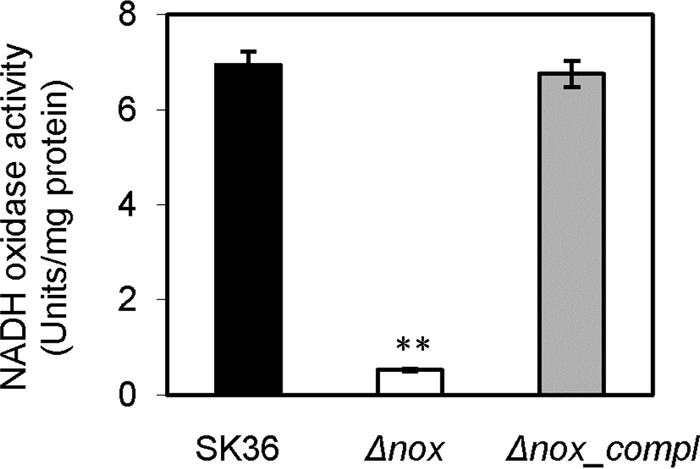
Decrease in NADH oxidase activity in the *nox* mutant. Cell lysates were examined for NADH oxidase activity. Δ*nox*, the *nox* mutant; Δ*nox_compl*, the complemented strain of the *nox* mutant. **, *P* < 0.01. Data were obtained at least in biological triplicate.

To confirm the NADH oxidase activity of Nox in S. sanguinis cells, NADH oxidase activities were compared among the wild-type strain (SK36), the *nox* deletion mutant, and its complemented strain. Compared to that in the wild-type strain, NADH oxidase activity was dramatically decreased in lysates of the *nox* mutant and restored after complementation of the mutant (Fig. 3). This result suggests that the Nox protein possesses NADH oxidase activity and, moreover, is the primary NADH oxidase in S. sanguinis cells.

### Extracellular decrease and intracellular increase of H_2_O_2_ in the *nox* mutant.

**FIG 4:**
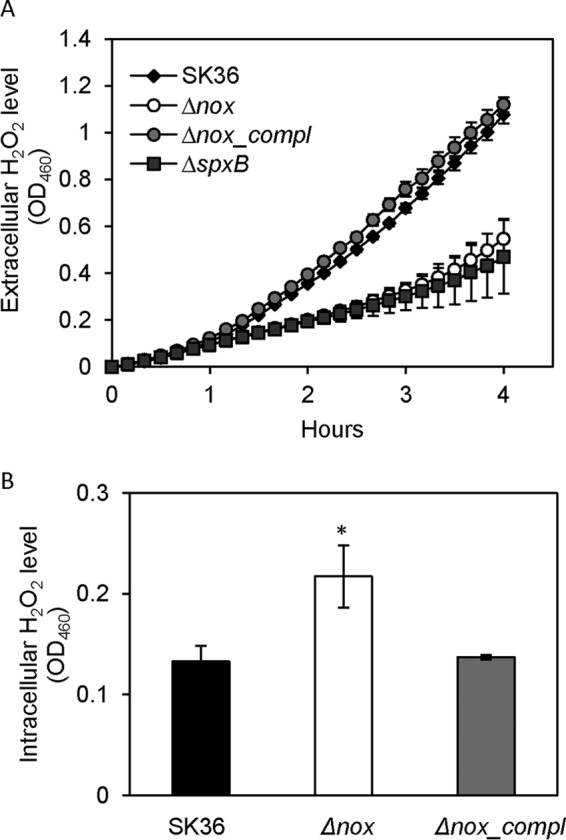
Extracellular (A) and intracellular (B) hydrogen peroxide production in the *nox* mutant. H_2_O_2_ production was measured in culture supernatants (A) or cell lysates (B) of cultures containing SK36 and mutant strains. Δ*nox*, the *nox* mutant; Δ*nox_compl*, the complemented strain of the *nox* mutant; Δ*spxB*, the *spxB* mutant. An asterisk indicates a significant difference at a *P* value of <0.05 compared to SK36. Data were obtained at least in biological triplicate.

To examine whether the *nox* gene was involved in H_2_O_2_ production in S. sanguinis cells, extracellular and intracellular H_2_O_2_ levels in the wild-type, *nox* mutant, and complemented strain were determined. The results showed the level of extracellular H_2_O_2_, detected using Amplex red, decreased in the *nox* mutant to the same level as that in a mutant deleted for the gene encoding the H_2_O_2_-producing pyruvate oxidase, SpxB. H_2_O_2_ levels in the *nox* complemented strain were the same as those of the wild type (Fig. 4A). Surprisingly, the level of H_2_O_2_ in an intracellular extract of *nox* mutant cells was significantly greater than that in the wild-type and complemented strains (Fig. 4B). Our previous study confirmed that SpxB is a major producer of H_2_O_2_ in S. sanguinis (17). However, the transcription of the *spxB* gene, assayed using qRT-PCR, and the SpxB activity in the *nox* mutant were not changed compared to that of the wild type and the complemented mutant (see Fig. S1 in the supplemental material).

### Involvement of the NADH oxidase gene in endocarditis virulence.

**FIG 5:**
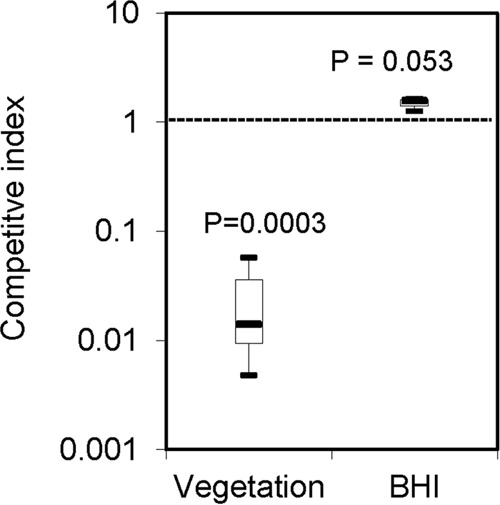
Attenuation in competitive index *in vivo* but not *in vitro* in the *nox* mutant. Vegetation, bacteria obtained from rabbit heart vegetation postinoculation; BHI, bacteria cultured in BHI broth. Data were obtained at least in biological triplicate.

S. sanguinis is one of most common causes of bacterial endocarditis. A number of virulence factors for endocarditis have been identified in S. sanguinis (26–29). After the inoculation of precatheterized rabbits with a 1:1 mixture of the *nox* mutant and the wild type, followed by overnight incubation, the CI of the *nox* mutant relative to that of the wild type in the infected vegetation was measured. As shown in Fig. 5, the CI in the vegetation was 0.016, which was significantly less than 1 (*P* = 0.0003), indicating the reduced fitness of the mutant. To confirm the reduced fitness of the mutant was not due to a general growth deficiency, we also performed competitions with the mutant and the wild type in BHI medium (i.e., *in vitro*). The CI of the *nox* mutant compared to that of the wild type was not significantly different from 1 after the incubation of the mixed inoculum in BHI medium overnight (Fig. 5). This suggested that there was no growth difference between the mutant and the wild type. These data indicated that the virulence of the *nox* mutant was impaired and that the *nox* gene played a role in IE.

### Blood killing of the *nox* mutant.

**FIG 6:**
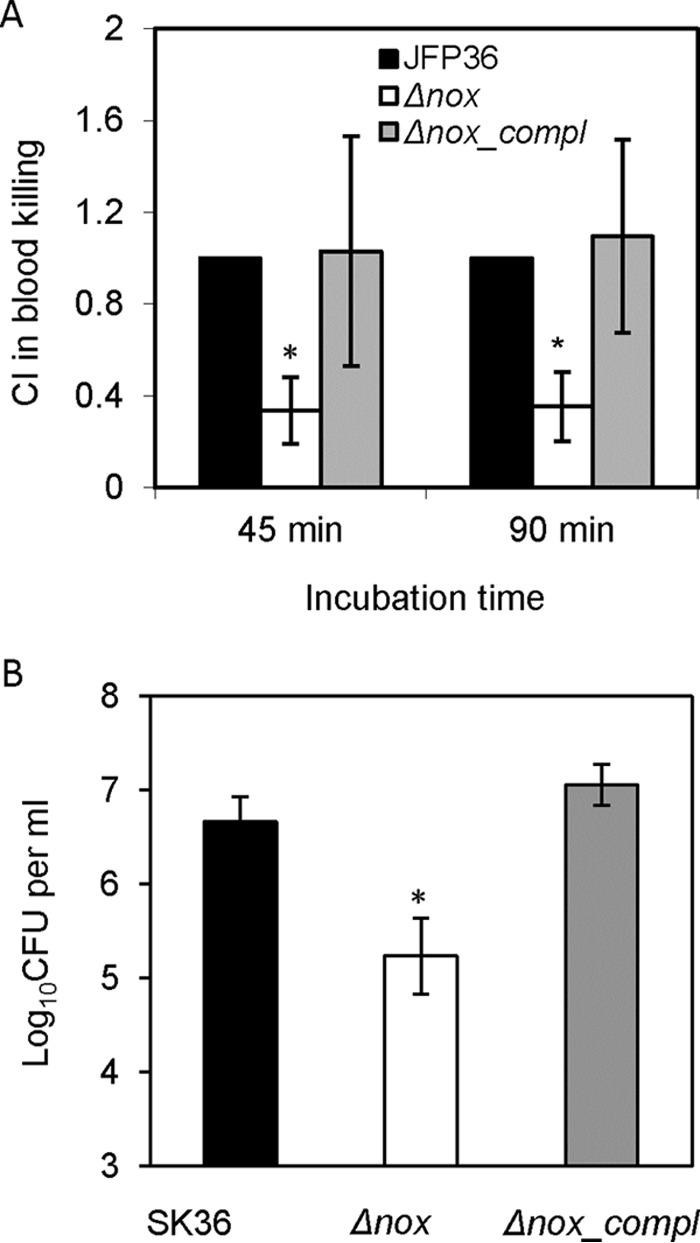
Decrease in blood killing (A) and growth in human serum (B) in the *nox* mutant. JFP36, an erythromycin-resistant derivative of SK36; Δ*nox*, the *nox* mutant; Δ*nox_compl*, the complemented strain of the *nox* mutant. An asterisk indicates significant difference at a *P* value of <0.05 compared to SK36 or JFP36. Data were obtained at least in biological triplicate.

Survival of the *nox* mutant compared to that of the wild type was examined in human blood. As shown in Fig. 6A, the CI of the *nox* mutant compared to that of the wild type was significantly less than 1 after 45 min and 90 min of incubation in blood. The complementation of the *nox* mutant restored survival to wild-type levels. These data indicated that the deletion of the *nox* gene gave rise to the decreased survival of S. sanguinis in human blood.

### Reduced growth of the *nox* mutant in serum.

The growth of the *nox* mutant in human serum was compared to that of the wild type. The results showed that the *nox* mutant was recovered in lower numbers than the wild type from human serum after overnight growth under microaerobic conditions (Fig. 6B). Thus, *nox* is required for the normal growth of S. sanguinis under *in vivo*-like conditions.

### Sensitivity of the *nox* mutant to exogenous H_2_O_2_ and acid.

**FIG 7:**
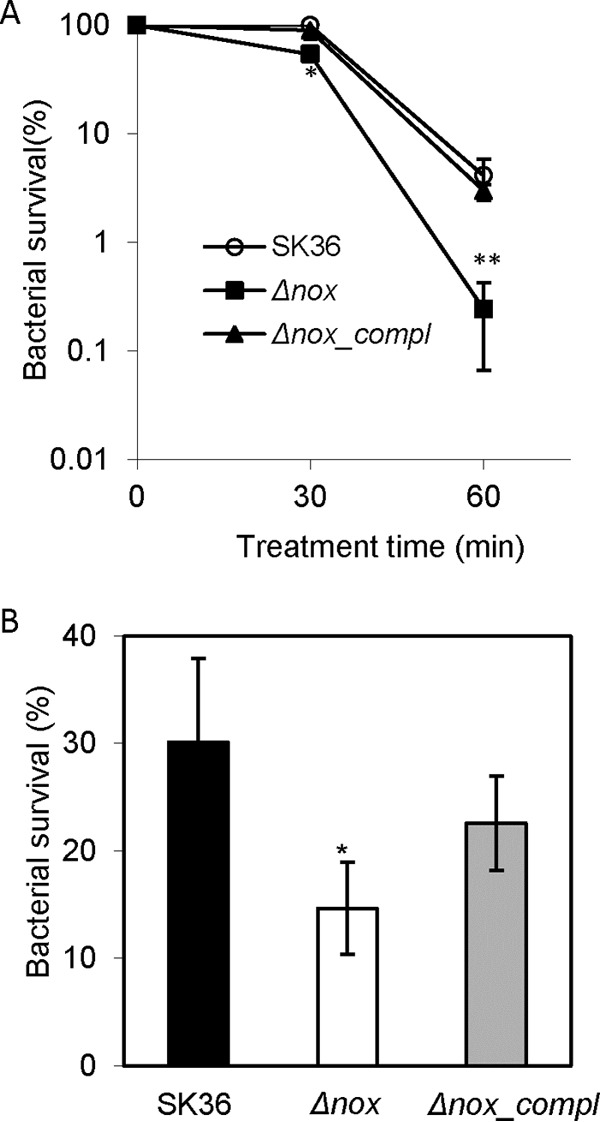
Sensitivity to environmental stresses in the *nox* mutant. Reduction in bacterial survival upon exposure to exogenous H_2_O_2_ (A) and acid (B) in the *nox* mutant. Δ*nox*, the *nox* mutant; Δ*nox_compl*, the complemented strain of the *nox* mutant. An asterisk indicates a significant difference at a *P* value of <0.05 compared to SK36. **, *P* < 0.01. Data were obtained at least in biological triplicate.

Neutrophil oxidative burst and acidification of phagosomes have been implicated in the bactericidal function of phagocytes (30); therefore, the sensitivity of the *nox* mutant to exogenous H_2_O_2_ and acid was examined. With H_2_O_2_ treatment, the survival of the *nox* mutant was markedly reduced after 1 h compared to that of the wild type. Complementation of the *nox* mutant restored survival to the same level as that of the wild type (Fig. 7A). Upon acid treatment, survival of the *nox* mutant also exhibited a significant decrease compared to that of the wild type (Fig. 7B). These data indicated that the *nox* mutant was more sensitive to H_2_O_2_ and acid stresses than the wild type.

## DISCUSSION

The *nox* orthologs have been demonstrated to encode NADH oxidase in S. pneumoniae (4), S. mutans (2), and S. agalactiae (1). This NADH oxidase is proposed to produce H_2_O from O_2_ (1, 2, 4), but this has not been confirmed. In this study, the S. sanguinis nox mutant exhibited a dramatic reduction in NADH oxidase activity compared to that of the wild type (Fig. 3), and rNox also exhibited NADH oxidase activity (Fig. 2A). Furthermore, the lack of H_2_O_2_ formation from O_2_ by rNox also was demonstrated (Fig. 2B). These data indicate that S. sanguinis Nox does indeed function as an H_2_O-forming NADH oxidase in the presence of oxygen.

It is interesting that the *nox* deletion decreased the extracellular H_2_O_2_ level (Fig. 4A) but increased the intracellular H_2_O_2_ level (Fig. 4B). The expression of the *spxB* gene and H_2_O_2_-producing activity of its gene product did not change in the *nox* mutant compared to that of the wild type (see Fig. S1 in the supplemental material), suggesting the increase in intracellular H_2_O_2_ level is not caused by enhancing the activity of the H_2_O_2_ producer. In E. coli, two scavenging enzymes, alkyl hydroperoxide reductase and catalase, were responsible for scavenging intracellular H_2_O_2_ (31). The mutation of either one could cause intracellular H_2_O_2_ to be elevated. Although intracellular H_2_O_2_ could penetrate the membrane to exit the cell, no H_2_O_2_ escaped from E. coli cells in the presence of these two enzymes. However, there are no homologs of these genes in the S. sanguinis genome, and S. sanguinis has been demonstrated to secrete H_2_O_2_ to inhibit S. mutans growth (16, 17). Here, we also showed the similar antagonistic results for S. sanguinis wild-type strain SK36 against S. mutans (Fig. 1 and 4A). These findings suggest S. sanguinis keeps the endogenous H_2_O_2_ level balanced through efflux instead of scavenging enzymes. In addition, we found the *nox* mutant failed to inhibit the growth of S. mutans (Fig. 1), further indicating extracellular H_2_O_2_ was decreased. In another study, we report a reduction in membrane fluidity in the *nox* mutant and do not find peroxidase-like genes up- or downregulated in the expression profiling of the mutant (38). It has been demonstrated that the permeation of H_2_O_2_ across biomembranes is rapid but limited (32), and that the E. coli membrane is semipermeable to H_2_O_2_ in cells (31). Therefore, it is possible that the reduction in membrane fluidity influences the diffusion of H_2_O_2_ across cell membranes, which leads to a decrease in extracellular H_2_O_2_ and increase in intracellular H_2_O_2_ in the *nox* mutant.

NADH oxidase has been documented to be involved in virulence in other streptococci. A *nox* insertion or deletion mutant was found to be significantly attenuated for the virulence of S. pneumoniae in an intraperitoneal model of sepsis in BALB/c mice (4, 33), a murine respiratory tract infection model, and a Mongolian gerbil otitis media infection model (34). Yamamoto et al. found significant attenuation in the virulence of S. agalactiae in lung, intraperitoneal, and intravenous murine infection models in the *nox* mutant (1). In this study, we found that the virulence of S. sanguinis in the rabbit endocarditis model was attenuated by the deletion of *nox* (Fig. 5), implicating *nox* in the virulence of S. sanguinis in IE.

Our study found the survival of the S. sanguinis nox mutant was significantly decreased in human blood (Fig. 6A) as well as human serum (Fig. 6B). The survival of the *nox* mutant also was diminished upon exposure to exogenous H_2_O_2_ or acid (Fig. 7). The neutrophil oxidative burst, which generates reactive oxygen species, and acidification of phagosomes have been proposed to play pivotal roles in the bactericidal function of phagocytes (35, 36). Therefore, these results suggest that the decreased survival of the *nox* mutant in blood is one of the reasons for attenuation in the virulence of S. sanguinis for IE, and that the decreased ability of the *nox* mutant to survive in human blood may be caused by both the growth reduction in human serum and greater sensitivity to neutrophil oxidative burst and acidification of phagosomes. Another possibility is that the higher intracellular H_2_O_2_ level may render the *nox* mutant more sensitive to H_2_O_2_ or other stresses.

Overall, our study found an S. sanguinis nox mutant failed to inhibit the growth of S. mutans, which may be caused by a decrease in H_2_O_2_ release from the bacterial cells. The mutation of the *nox* gene also attenuated the virulence of S. sanguinis in IE. This may be associated with decreased survival/growth in blood and serum and more sensitivity to acid and oxidative stresses. Since the *nox* gene is widespread in other species of streptococci, continuation of this work may lead to a comprehensive elucidation of the underlying mechanisms of competition and virulence for streptococci.

## Supplementary Material

Supplemental material
