# Supplementary material for: Involvement of NADH Oxidase in Competition and Endocarditis Virulence in Streptococcus sanguinis
Source: Infect Immun. 2016 Apr 22;84(5):1470–7. doi: 10.1128/IAI.01203-15 (PMC4862721; doi:10.1128/IAI.01203-15)
Supplement: Supplemental material [file supp_84_5_1470__index.html]

Supplemental material 

# Involvement of NADH Oxidase in Competition and Endocarditis Virulence in Streptococcus sanguinis

## Supplemental material

- Supplemental file 1 -

  Fig. S1. Expression of the *spxB* gene determined via qRT-PCR and pyruvate oxidase (SpxB) activity in the *nox* mutant cultured under microaerobic conditions.

  PDF, 103K
