# Supplementary material for: Involvement of NADH Oxidase in Competition and Endocarditis Virulence in Streptococcus sanguinis
Source: Infect Immun. 2016 Apr 22;84(5):1470–7. doi: 10.1128/IAI.01203-15 (PMC4862721; doi:10.1128/IAI.01203-15)
Supplement: Supplemental material [file IAI.01203-15_zii999091691so1.pdf]

Supplemental Material

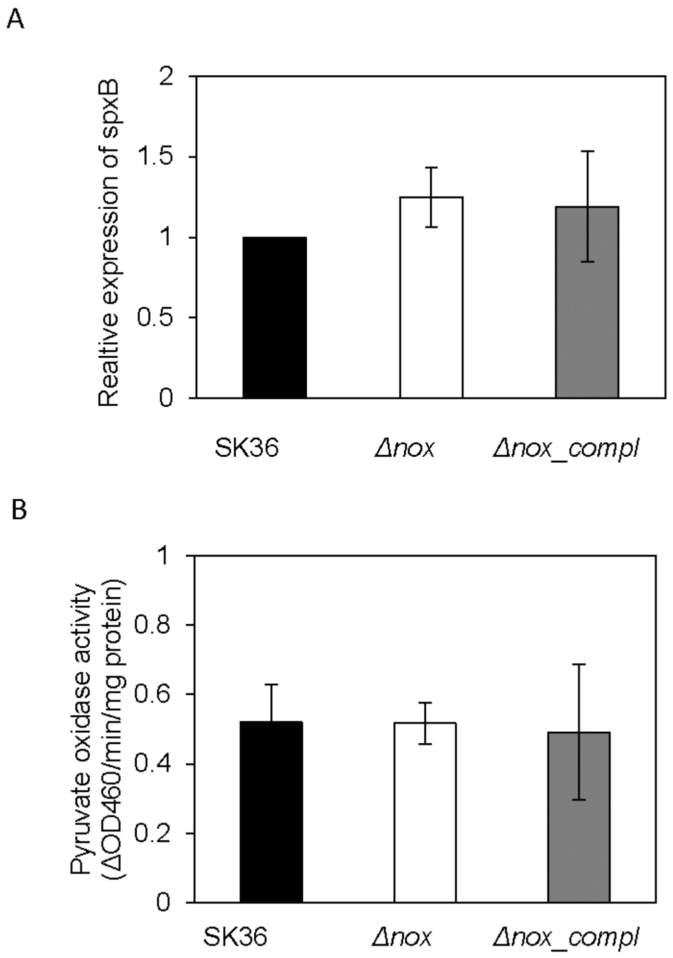

Fig. S1. Expression of the *spxB* gene using qRT-PCR (A) and pyruvate oxidase (SpxB) activity (B) in the *nox* mutant cultured under microaerobic conditions.
